# Whole exome sequencing identifies a novel *EMD* mutation in a Chinese family with dilated cardiomyopathy

**DOI:** 10.1186/1471-2350-15-77

**Published:** 2014-07-05

**Authors:** Mingqiu Zhang, Jia Chen, Dayong Si, Yu Zheng, Haixu Jiao, Zhaohui Feng, Zhengmao Hu, Ranhui Duan

**Affiliations:** 1 State Key Lab of Medical Genetics, Central South University, 410008 Changsha, Hunan province, China; 2 China Japan Union Hospital, Jilin University, 130031 Changchun, Jilin province, China; 3 School of Life Science, Jilin University, 130031 Changchun, Jilin province, China

**Keywords:** Dilated cardiomyopathy, Emery-Dreifuss muscular dystrophy, *EMD* gene, Novel mutation, Whole exome sequencing

## Abstract

**Background:**

Variants in the emerin gene (*EMD*) were implicated in X-linked recessive Emery-Dreifuss muscular dystrophy (EDMD), characterized by early-onset contractures of tendons, progressive muscular weakness and cardiomyopathy. To date, 223 mutations have been reported in *EMD* gene and the majority of them caused a predominant skeletal muscular phenotype. In this study, we identified a novel deletion mutation in *EMD* exon 1, which results in almost a complete loss of emerin protein in a large Chinese family. However, the patients suffered severe dilated cardiomyopathy (DCM) but very mild skeletal muscle disorder.

**Case presentation:**

Whole exome sequencing (WES) and linkage analysis were performed to identify the underlying mutation in a Chinese DCM family spanning five generations. A missense variation in the *GPR50* gene was found co-segregated with the disease phenotype, whereas no functional alteration was detected in the variant GPR50 protein. When analyzing the failure sequences in the exome sequencing data, a novel deletion mutation (c.26_39delATACCGAGCTGACC) in *EMD* exon 1, was identified in this family. Different from the typical clinical features caused by most reported *EMD* mutations, patients in our study presented very mild skeletal muscle degeneration that had not been diagnosed until the mutation was found.

**Conclusion:**

We described a family with rare clinical presentations caused by a novel *EMD* deletion mutation. Our findings broaden the heterogeneous spectrum of phenotypes attributed to *EMD* mutations and provide new insight to explain the genotype-phenotype correlations between *EMD* mutations and EDMD symptoms.

## Background

The *EMD* gene, consisting of 6 exons and encoding a nuclear envelope protein emerin (254 amino acids), was involved in X-linked Emery-Dreifuss muscular dystrophy (EDMD) [1]. Among the 223 reported *EMD* mutations in the Leiden Open Variation Database (LOVD), the overwhelming majority of mutations have been identified in EDMD patients who presented childhood-onset contractures of the elbows, Achilles tendons and post-cervical muscles, slowly progressive muscle weakness with humeroperoneal distribution in adolescence and cardiomyopathy with conduction block in early adulthood [1,2].

Dilated cardiomyopathy (DCM) is a common cause of advanced heart failure and the primary indication for heart transplantation [3]. Currently over 40 individual genes that encode for sarcomeric, cytoskeletal, nuclear membrane, dystrophin-associated glycoprotein complex and desmosomal proteins have been implicated in inherited DCM [3,4]. One of the most common nonsyndromic DCM-causing genes is *LMNA*, which encodes the nuclear lamina proteins lamin A and C; mutations in this gene also cause autosomal dominant and recessive EDMD [1,4]. Even though the functions of emerin and lamin A, C are very similar, the *EMD* gene is not listed in the routine screening of DCM because of lacking sufficient body evidences [4].

In this study, we conducted WES combined with linkage analysis to identify the underlying mutation in a large Chinese DCM family. A missense variation in the *GPR50* gene was found co-segregated with the disease phenotype, whereas no functional alteration was detected in the variant GPR50 protein. When analyzing the failure sequences in the WES data, a novel deletion mutation (c.26_39delATACCGAGCTGACC) in *EMD* exon1, which results in almost a complete loss of emerin protein, was identified in this family. Different from the typical clinical features caused by most reported *EMD* mutations, the patients in our study presented severe DCM but very mild skeletal atrophy.

## Case presentation

### Materials and methods

#### Subjects

**Figure 1:**
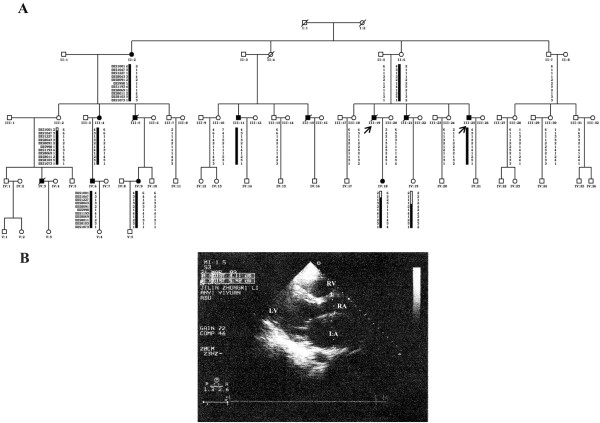
**Haplotype analysis of family pedigree and ultrasonic cardiogram (UCG) study of proband A. (A)** All sampled subjects in the pedigree are identified by their Roman numerals below the symbol. Arabic numerals denote each individual in a generation. Open symbols = unaffected; filled symbols = affected; symbols with a diagonal line = deceased subjects; squares = male; circles = female; arrows = the probands; Haplotype analysis is shown for chromosome X using 11 markers. The black bar indicates the haplotype assumed to carry the disease allele. The bars with black and white haplotypes indicate the existence of recombination event. The minimal in-linkage interval is 6.2 Mb of genomic DNA, 20.9 cM in size between DXS8091 and DXS1073 on chromosome Xq28. **(B)** Ultrasonic cardiogram study of proband A shows chamber enlargement. LA = left atrium; LV = left ventricle; RA = right atrium; RV = right ventricle.

A pedigree from Jilin Province, China, consists of 73 family members (40 males and 33 females) in five generations (Figure 1A). Familial DCM was diagnosed according to WHO1995 diagnostic criteria (left ventricle end diastolic diameter > 2.7 cm/m^2^; fractional shortening < 25%). The study was approved by the expert committee of hospital of the Jilin University in China (equivalent to an institutional review board).

### Linkage analysis

Peripheral blood of 39 family members was obtained. Genomic DNA was extracted from the peripheral blood lymphocytes, using the standard phenol–chloroform method. A total of 22 subjects were included in the linkage study and followed fine mapping analysis. Genotyping was performed using 18 microsatellite markers (Panel 28, Version 2.5, ABI PRISM Linkage Mapping Set) in X chromosome. Five additional markers (DXS998, DXS1193, DXS8069, DXS8011, DXS8103) were chosen to refine mapping the gene locus. Two-point LOD scores were calculated by the program MLINK, and multipoint LOD scores with use of Linkmap. The disease was considered to be X-linked with a frequency of 0.0001 and 95% penetrance.

### Exome sequencing

Five micrograms of DNA from each of two affected male individuals was applied for the construction of exome library using the Agilent SureSelect exome capture system and was sequenced on the Illumina Solexa GAIIx platform following the manufacturer’s instructions (Illumina, San Diego, CA). Raw image files were processed by the Illumina pipeline (version 1.3.4) for base calling and generating the reads set.

Reads were aligned to the human reference genome (University of California Santa Cruz, UCSC hg19) using SOAPaligner. Single nucleotide polymorphisms (SNPs) and indels (insertions and deletions) identification was performed by samtools and/or Genome Analysis Toolkit (GATK) and such SNPs with read depth > 4 and quality > 20 were reserved for subsequent analysis. SNPs and indels were annotated using SeattleSeq annotation. On the basis of SNP database (dbSNP) and 1000 genomes annotation and the supplied PolyPhen prediction, any non-synonymous variants not assigned a ‘benign’ prediction were considered to be damaging.

### Analysis of candidate genes

DNA sequences were obtained from GenBank. Primers were designed with primer-3 software. Protein-encoding exons of candidate genes were amplified from genomic DNA. PCR products were purified with use of kits (golden polymerase, ABI, California) and sequenced with a dye-terminator cycle-sequencing system (Applied Biosystems, California).

## Results

### Clinical features

All the individuals in the family were interviewed, and reported symptoms of cardiomyopathy disease such as chest pain, shortness of breath and fatigue; 8 males and 4 females were identified with clinical symptoms (Figure 1A). Sudden cardiac death was reported in five of eight male patients at the age of 25, 30, 33, 33 and 42 years old. The female patients presented milder heart failure and less life-threatening symptoms than the males. The proband A (III: 19) was initially diagnosed as sick sinus syndrome with chest tightness and early onset fatigue, especially after heavy activity, and his electrocardiogram (ECG) showed sinus bradycardia (sinus node dysfunction), with a minimum heart rate of 36 times/min at the age of 17 years. The symptoms progressively became more severe when the patient was 34 years old. The pacemaker implantation failed and the ultrasonic cardiogram (UCG) showed chamber enlargement (left ventricular end-diastolic diameter of 56.8 mm, the right atrium 65 × 83 mm, right room 26.4 mm, left atrial 54.2 mm) (Figure 1B), with a thinning of the chamber wall. The subject was hospitalized at the age of 41 years; the 24-h Holter detected paroxysmal ventricular tachycardia and sudden death occurred 1 month after discharge. A similar cardiac disorder has been identified in other patients in the family, but not among unaffected members.

### Candidate genes analysis and Linkage study

Genetic and clinical evaluation of the pedigree provides the evidence of an X-linked recessive inheritance. *DMD* and *TAZ* are the two reported genes associated with X-linked nonsyndromic DCM [3]. No deletion or insertion was observed in the *DMD* gene of proband A using the method of multiplex ligation-dependent probe amplification (MLPA) and Sanger sequencing did not detect any mutation in the exons and exon-intron junctions of both strands in the *TAZ* gene. Linkage analyses in the family identified a locus associated with DCM on chromosome X, band Xq28 (maximal lod score 1.55 at DXS1073). Five additional markers were analyzed to narrow the positive candidate region, defined a common haplotype in all the affected subjects, yielding a maximum 2-point LOD score of 2.43 with a marker located at DXS1193 (Figure 1A).

### Whole exome sequencing

Whole exomes of two affected male individuals in the family (sample III: 11 and IV: 6) were sequenced, generating an average of 4.5 billion bases of sequence and a mean coverage of 65× for each affected individual. An average of 97% of the targeted bases was sufficiently covered to pass our thresholds for variant calling. Variants of 73 hereditary cardiomyopathies related genes were screened [5], the filtering conditions are as following: (1) same variants in both WES data; (2) missense, nonsense, insertion and deletion variants; (3) SNPs with minor allele frequency not more than 0.005 according to the SNP database of National Center for Biotechnology Information (NCBI). 7 variants passed the filtering conditions and none of them co-segregated with the disease phenotype. Then 140 genes in the linked locus (Xq28) were also screened according to the above conditions. 9 variants passed the conditions and only the *GPR50* c.113C > T variant was found co-segregation with the disease phenotype.

### Candidate variant analysis

**Figure 2:**
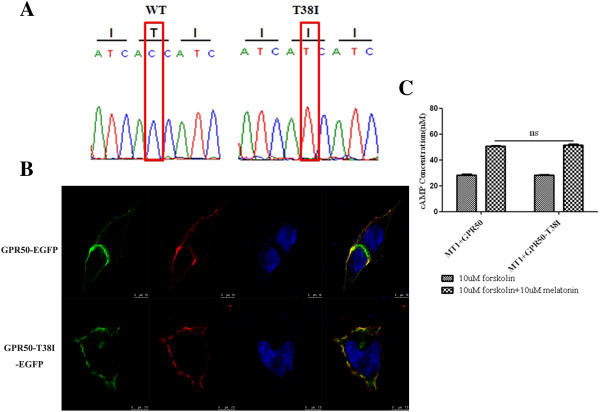
**Identification and functional study of the *GPR50 *variant. (A)** Sanger sequence of codons 37-39 in exon 1 of *GPR50* in a wild-type (WT) subject and proband B confirms the presence of the T38I variation. The variant nucleotide residue is indicated by a red rectangular. **(B)** The wild-type and variant GPR50 protein both located on the membrane of HEK293 cells. GFP-tagged GPR50 wild-type and variant plasmids were transfected into HEK293 cells. The red plasma membranes were labeled by Concanavalin A (Con A) and the nuclei were stained blue with DAPI. **(C)** The variant GPR50 protein did not alter the effect on melatonin-induced MT1 signaling. GPR50 wild-type and variant plasmids were transfected into MT1 stable transfection HEK293 cells, no difference of cAMP levels was deteced when treated with forskolin and melatonin, 48 h after trnasfection (ns, P > 0.05).

GPR50 is a member of G-protein coupled receptor 1 family and known as a melatonin-related orphan receptor. The physiological functions of this protein are still largely unknown [6]. The variant c.113C > T (rs189225995) in *GPR50* was tested in 1500 controls (314 males and 593 females, normal unaffected individuals of matched geographical ancestry) and found in 2 females. The substitution of polar Thr to nonpolar Ile caused by the variant in the first transmembrane domain of GPR50 did not alter the three-dimensional structure when predicted using the Phyre2 server and seven temples (SCOP codes: c2ksaA [human Substance-P receptor], c3rzeA [Histamine H_1_ receptor], c2rh1A [β_2_ adrenergic receptor], c3emlA [Human Adenosine A2A receptor], c4djhA [human j-type opioid receptor], c3uonA [human M2 muscarinic acetylcholine receptor], c3pdsA [human b2-adrenergic receptor]). Further, both the variant and wildtype GPR50 protein could locate on the plasma membrane when transfected into HEK293 cells (Figure 2B). GPR50 could alter melatonin-induced MT1 signaling, which couples the Gi protein inhibiting phosopholipase C activation and decreases cAMP levels through heterodimerization [6]; however, no apparent variation in cAMP signalling was detected when the variant and wildtype GPR50 protein were co-transfected with MT1 in HEK293 cells (Figure 2C). These experiments suggest that the variant is unlikely to be sufficient to cause DCM in the pedigree.

### Mutation detection and proband reexamination

**Figure 3:**
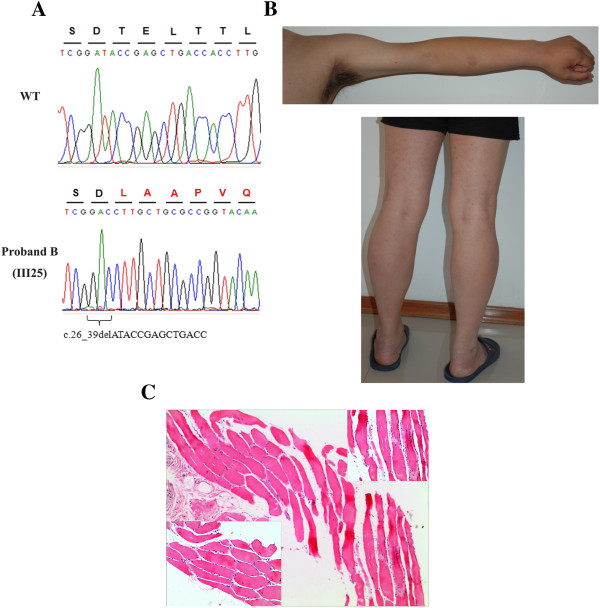
**The proband B carrying a novel deletion mutation in *EMD *presented mild skeletal disorder. (A)** Sanger sequence of codons 8-15 in exon 1 of *EMD* in a wild-type subject and proband B confirms the 14-bp deletion mutation (c.26_39delATACCGAGCTGACC). **(B)** A mild atrophy was observed in the proband B’s bicep and tricep muscles on the arm, no contraction had been detected in his elbows and Achilles tendons, and his calf muscles were surprisingly strong. **(C)** Hematoxylin-eosin staining of deltoid biopsy from proband B showed clear cross striation and normal myofilament fibers. Internally located nuclei and fiber splitting were found. Endomysial fibrosis and sarcoplasmic condensation were occasionally noted.

Further analyzing the WES data revealed that twenty exons of 10 genes on the linked locus were demonstrated sequencing failure due to a high GC-content. Sanger sequencing was performed to screen these exons. A 14-bp deletion mutation (c.26_39delATACCGAGCTGACC) in the *EMD* exon 1, with no record in the LOVD, causing a frameshift and a premature stop codon at position 81, and generating a truncated 26-amino acid polypeptide, was identified in all the patients and absent in unaffected family members (Figure 3A). Considering that most mutations in the *EMD* caused severe skeletal muscle disorder, the proband B (III: 25) who had suffered from sinus bradycardia with a minimum heart rate of 42 times/min was reexamined. A mild atrophy was observed in the patient’s bicep and tricep muscles on the arms (Figure 3B), and a gentle contraction was detected in his spinal column. However, no contraction had been detected in his elbows and Achilles tendons, and weakness or atrophy was not observed in his calf muscles, which were surprisingly as strong as unaffected family members (Figure 3B).The creatine kinase (CK) level of proband B was elevated at 442 U/L (normal = 24-190 U/L). The electromyography (EMG) evaluation identified myogenic damage. The amplitude and duration of motor unit potentials in right biceps brachii and right deltoid were reduced, but no obvious abnormality was found in both sides of gastrocnemius muscle. The conduction velocity of motor and sensory fibers was normal. The biopsy of deltoid showed clear cross striation and normal myofilament fibers. Internally located nuclei and fiber splitting were found. Endomysial fibrosis and sarcoplasmic condensation were occasionally noted (Figure 3C).

## Discussion

In this study, we performed WES and linkage analysis to identify a novel mutation in the *EMD* gene in a large Chinese DCM family. The WES ruled out the mutations in the exons of all reported nonsyndromic DCM-causing genes and found a single-nucleotide variant in *GPR50* gene which was completely co-segregated with the disease phenotype. But the following functional studies did not reveal any functional change between the wild-type and variant GPR50 protein. These results pushed us to screen the sequencing failure exons in the WES data and eventually the disease-causing mutation in *EMD* was found. This process highlighted the indispensability of functional study in identifying new disease-causing genes and the importance of analyzing the sequencing failure data of WES.

Most reported mutations in the *EMD* gene caused apparent skeletal muscle disorders with cardiomyopathy in the form of EDMD [1,2]. A novel deletion mutation in *EMD* exon 1 which results in almost a complete loss of emerin protein was identified in the pedigree. However, different from the typical EDMD clinical features caused by most reported *EMD* mutations, the patients in our study did not present apparent skeletal muscle atrophy and contractures. To date, only three *EMD* mutations (an in-frame 3-bp deletion in exon 2, a 5-bp deletion and a nonsense mutation in exon 6) were reported in patients with predominant cardiac diseases and mild skeletal muscle disorder [7-9]. These cases together with our findings pointed out that environmental or genetic modifications, such as functional overlapping proteins complement, may contribute to the observed clinical variability caused by *EMD* mutations.

Emerin is a member of the LEM-domain protein family, which presents a group of inner nuclear membrane and intranuclear proteins such as lamina-associated polypeptide 2 (LAP2) and MAN antigen 1 (MAN1) [1]. Key components of the Rb pathway, which plays a crucial role in E2F-mediated cell cycle regulation and MyoD-mediated induction of myogenesis, were specifically altered in human muscle biopsies of EDMD patients [10]. Emerin-knockout mice also revealed that Rb1 and MyoD expression levels and pathways during muscle regeneration were disrupted even though no overt abnormality was observed [11]. Consistently, the protein levels of MyoD decreased in the complete and muscle-specific knockout mice of LAP2α, which is a mammal-specific non-membrane-associated isoform of the LAP2 gene [12]. A mutation in the C-terminal tail of LAP2α has also been linked to DCM [13]. These findings provided evidences that emerin and LAP2α may have some overlapping functions in the process of muscle regeneration and differentiation. Considering common cardiac disorder but diverse skeletal muscle involvement observed among the patients carrying *EMD* mutations, the functional complement from LAP2α and environmental effect may partly contribute to skeletal muscle regeneration.

## Conclusion

We applied WES and linkage analysis to identify a novel deletion mutation in *EMD* exon 1 (c.26_39delATACCGAGCTGACC) in a Chinese family spanning five generations. Different from the typical clinical features caused by the majority of reported *EMD* mutations, the patients in our study present severe DCM but very mild skeletal muscle disorder. Our study suggests the *EMD* gene, which was not listed in the routine screening of DCM, should be considered in patients with DCM of unknown etiology and further functional studies of emerin in skeletal and cardiac muscle cells are needed to illustrate the phenotypic heterogeneity caused by *EMD* mutations.

## Consent

Written informed consents including the publication of images in Figure 1 and Figure 3 were obtained from all participants or their legal representatives.

## Abbreviations

DCM: Dilated cardiomyopathy; WES: Whole exome sequencing; EDMD: Emery-Dreifuss muscular dystrophy; LOVD: Leiden open variation database; ECG: Electrocardiogram; UCG: Ultrasonic cardiogram; MLPA: Multiplex ligation-dependent probe amplification.

## Competing interests

The authors declare no competing of interest.

## Authors’ contributions

MZ was involved in the acquisition and analysis of clinical data. JC carried out the molecular genetic studies and participated in drafting the manuscript. DS participated in screening candidate genes. YZ contributed to analyze the whole exome sequencing data. HJ and ZF were involved in the collection of patients and in the acquisition and analysis of clinical data. RD and ZH conceived the study, participated in its design and coordination and contributed to draft the manuscript. All authors read and approved the final manuscript.

## Pre-publication history

The pre-publication history for this paper can be accessed here:

http://www.biomedcentral.com/1471-2350/15/77/prepub
